# Supplementary material for: Membrane bending occurs at all stages of clathrin-coat assembly and defines endocytic dynamics
Source: Nat Commun. 2018 Jan 29;9:419. doi: 10.1038/s41467-018-02818-8 (PMC5789089; doi:10.1038/s41467-018-02818-8)
Supplement: Supplementary file 1 — Supplementary Information [file 41467_2018_2818_MOESM1_ESM.pdf]

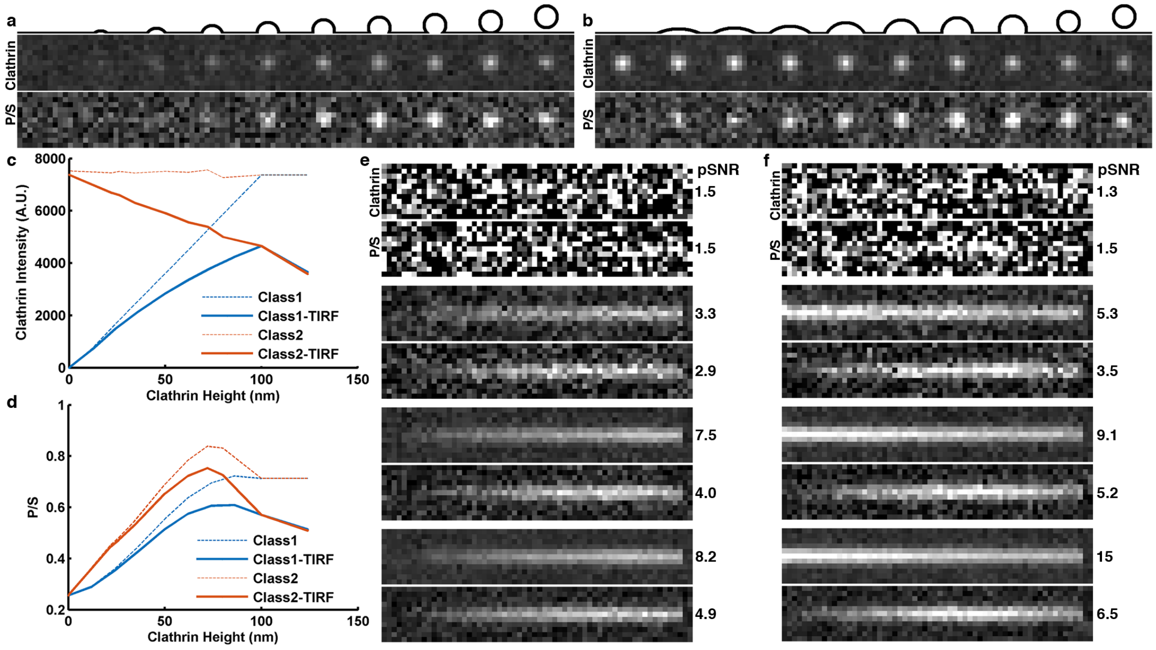

Supplementary Figure 1. Computer simulation for membrane bending during CME. a. Class 1 simulation; clathrin induces membrane deformation as it assembles until the fully formed vesicle departs from the plasma membrane. **b.** Class 2 simulation; clathrin assembled into a flat sheet which remodels into the final vesicle and departs from the plasma membrane. The evanescent exponentially decaying excitation field, orientation of DiI, and concentration of clathrin was accounted at each step of the progression with 2 nm resolution. Blurring from the optics was modeled using a Gaussian, and drawing intensity values from a Poisson function simulated detection noise. **c.** Integrated clathrin intensity of the high resolution simulation in the absence of noise, showing the effect of the evanescent field on each simulated class. **d.** Integrated intensity of P/S in the absence of noise. **e.** Class 1 kymographs with increasing peak SNR (pSNR). **f.** Class 2 kymographs with increasing pSNR. The pSNR was reported as the maximum SNR (μ/σ_{back}) calculated in the montage. The pSNR levels were varied to be above and below the average pSNR of clathrin-Tq2 (6.2) and P/S (3.4) from the dynamic imaging experiments. Images panels are 1µm on a side.

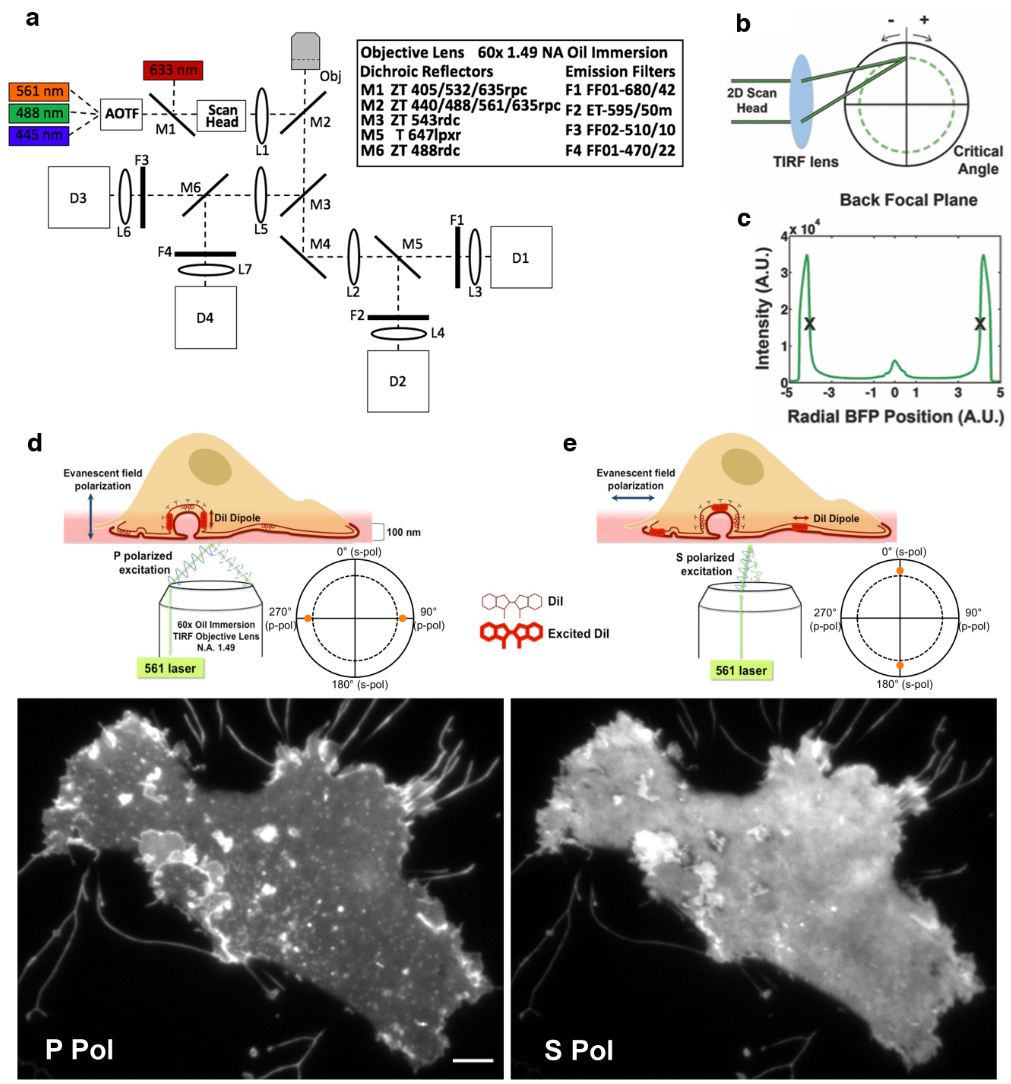

Supplementary Figure 2. polTIRF microscope schematic and polarization modulation. a. Schematic and list of the microscope's components (inset). **b.** Schematic of optical components for back focal plane positioning. A 2-dimensional scan head is used to position laser illumination at discrete positions in the back focal plane. **c.** Centering is quantified using measured intensities of reflected light at many positions in the BFP, projected onto a quadrant diode detector. Centering is defined as equal intensities at opposite, but equivalent positions of the BFP and is measured at full-width, half maximal positions, indicated by 'X' marks, on the plot. **d.** Back-focal plane positioning to generate orthogonal polarization. The 561 nm excitation was focused to the back focal plane at the defined positions. P polarized excitation was averaged from two-points 90˚ and 270˚ respectively. This preferentially excites vertically oriented DiI. **e.** S polarization was generated by excitation at 0˚ and 180˚ in the BFP to preferentially excite horizontal DiI. Scale bar is 5 μm.

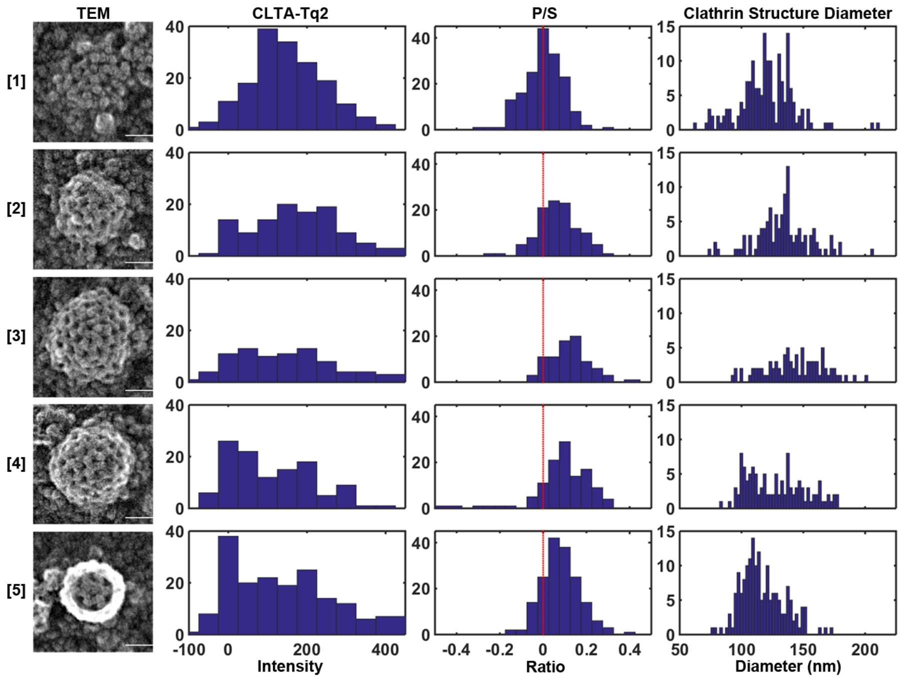

Supplementary Figure 3. Qualitative classification of EM data. Clathrin pit ultrastructure was classified into 5 categories based on increasing apparent curvature. [1] – Flat patch, [2] – Shallow curvature, [3] – Medium curvature, [4] – Domed curvature, [5] – Fully formed clathrin vesicle. The diameter of each clathrin object was quantified by drawing a circle centered on the object, the correlated clathrin-Tq2 fluorescence and P/S was quantified using the two ROI based approach, and the categorized data was plotted as a histogram. A dashed line is plotted at zero in the P/S histograms. Scale bars in TEM images are 50 nm.

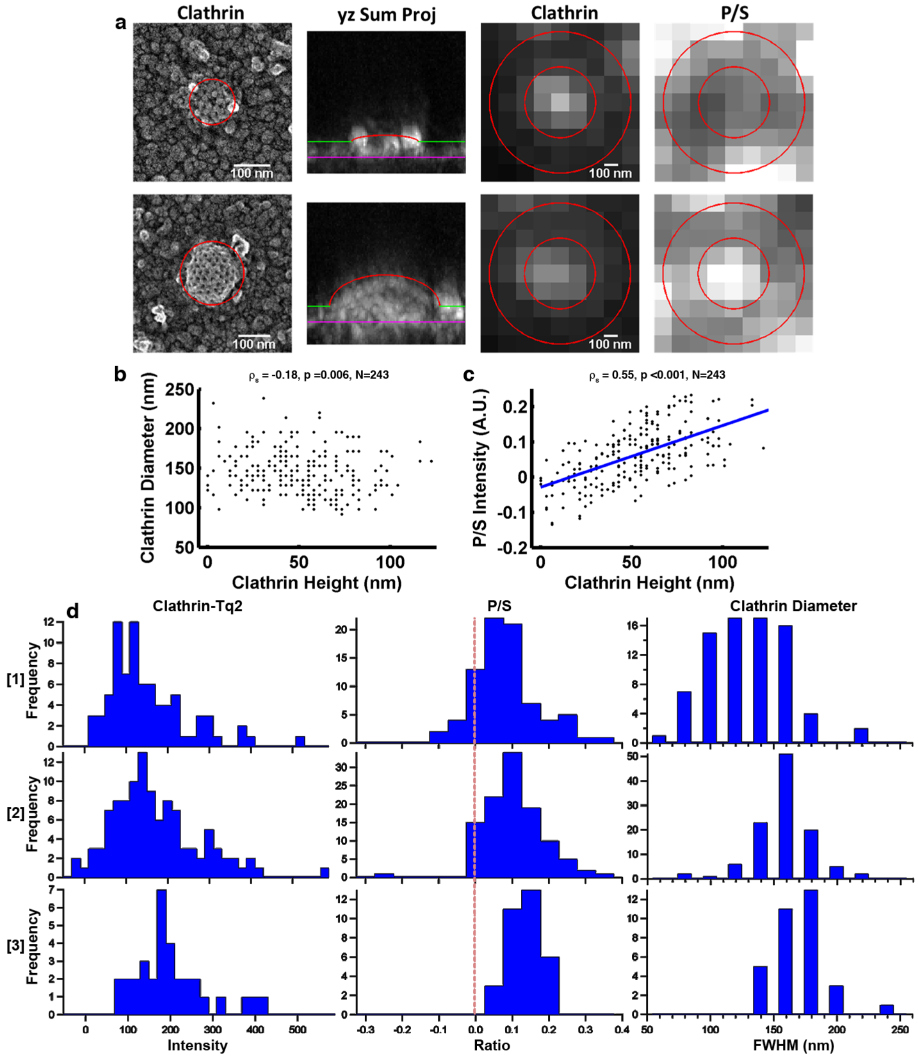

Supplementary Figure 4. Quantification of correlative heights and widths. a. Circles were drawn to surround the identified clathrin objects to measure the diameter. The height of the clathrin object was measured on the yz sum projection of the tilt-series tomogram. The correlated fluorescence intensities were quantified for clathrin-Tq2 and P/S. The signal (inner ROI) was corrected for local background by subtracting the outer ring ROI. **b.** Scatter plot of the clathrin diameter versus height measured from the tomogram, spearman correlation coefficient, and p value is shown. **c.** Correlative P/S Intensity and clathrin height, with spearman correlation coefficient and p value, and linear regression plot. **d.** Quantification of correlative AFM heights and widths with Tq2 fluorescence and P/S, with dashed line at zero for P/S. Clathrin objects were categorized by the measured AFM heights, [1]: <45 nm, [2]: 45-69 nm, and [3] >69 nm.

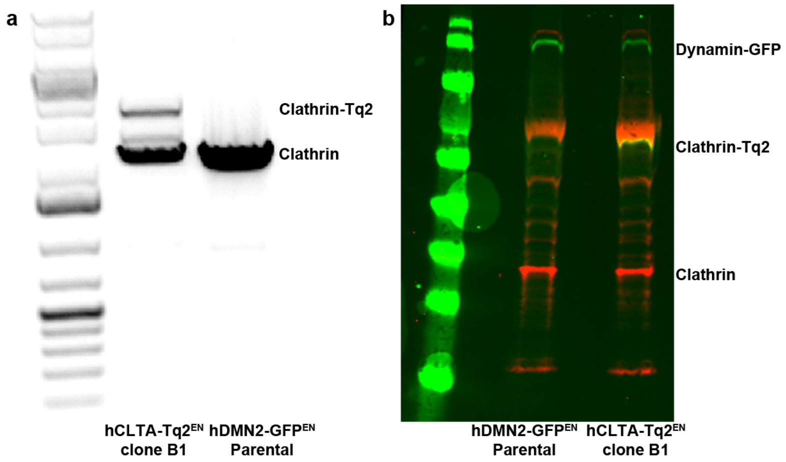

Supplementary Figure 5. Characterization of hCLTA-Tq2^{EN}/hDMN2-eGFP^{EN} SK-MEL-2 cell line. a. Out-out PCR demonstrating targeted insertion of mTq2 into the CLTA locus. Lane 1, 1kb plus ladder, Lane 2, hCLTA-Tq2^{EN} clone B1 and Lane 3, parental hDMN2-GFP^{EN}. **b**. Western blot analysis to confirm hCLTA-mTq2^{EN} fusion, Lane 1: Molecular weight ladder, Lane 2, Parental hDMN2-eGFP^{EN}, Lane 3, hCLTA-mTq2^{EN} clone B1. Overlay composite immunoblot for anti-clathrin light chain (red), and anti-GFP (green), which also cross-reacts with mTq2 resulting in the yellow band for hCLTA-mTq2^{EN}.

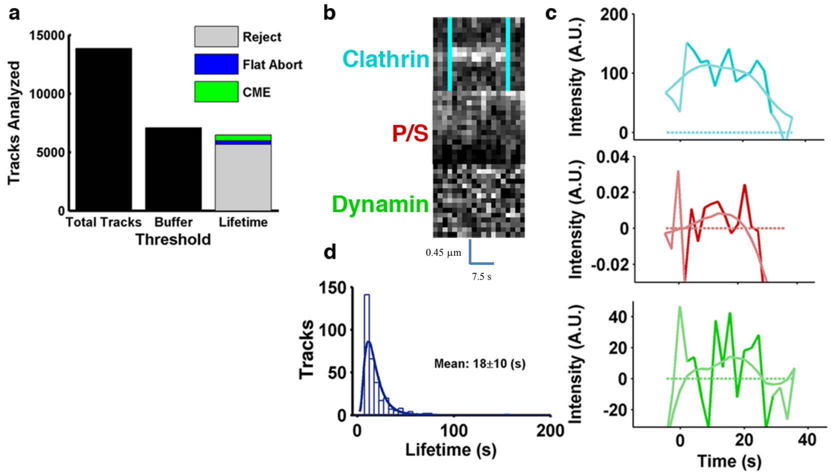

Supplementary Figure 6. Filtering criteria and analysis of abortive events. a. Data were pooled from 4 cells, and filtered based on the following criteria: Total tracks had a clathrin peak SNR of at least 3.5. Tracks were required to have 3 consecutive frames without a clathrin object before and after the track ('Buffer'). The tracks were also required to have a lifetime of at least 7 sec ('Lifetime'). The analyzed tracks are color coded as CME events (green), described in Fig 3, Flat abortive events, described in fig. S10, and tracks excluded from analysis (grey). The following reasons were the main causes of exclusion from analysis: 1) Clathrin objects that crossed paths with another track. 2) Events that were lacking either a dynamin or P/S signal. 3) Events where the P/S signal was occluded by additional areas of curvature not directly related to the endocytic event. **b.** Tracked and quantified results of a single clathrin assembly. Clathrin-Tq2 signals are labeled with cyan, P/S labeled in red, and dynamin-GFP labeled in green. The yt kymograph was made from the center column of pixels in the montage from the tracked spot, and the lifetime clathrin is indicated by the colored vertical bars. **c**. The local background corrected intensity for each signal over time. **d.** Lifetime histogram for the flat abortive events quantified across four cells, N=314. The average lifetime of these events was 18 ± 10 s.

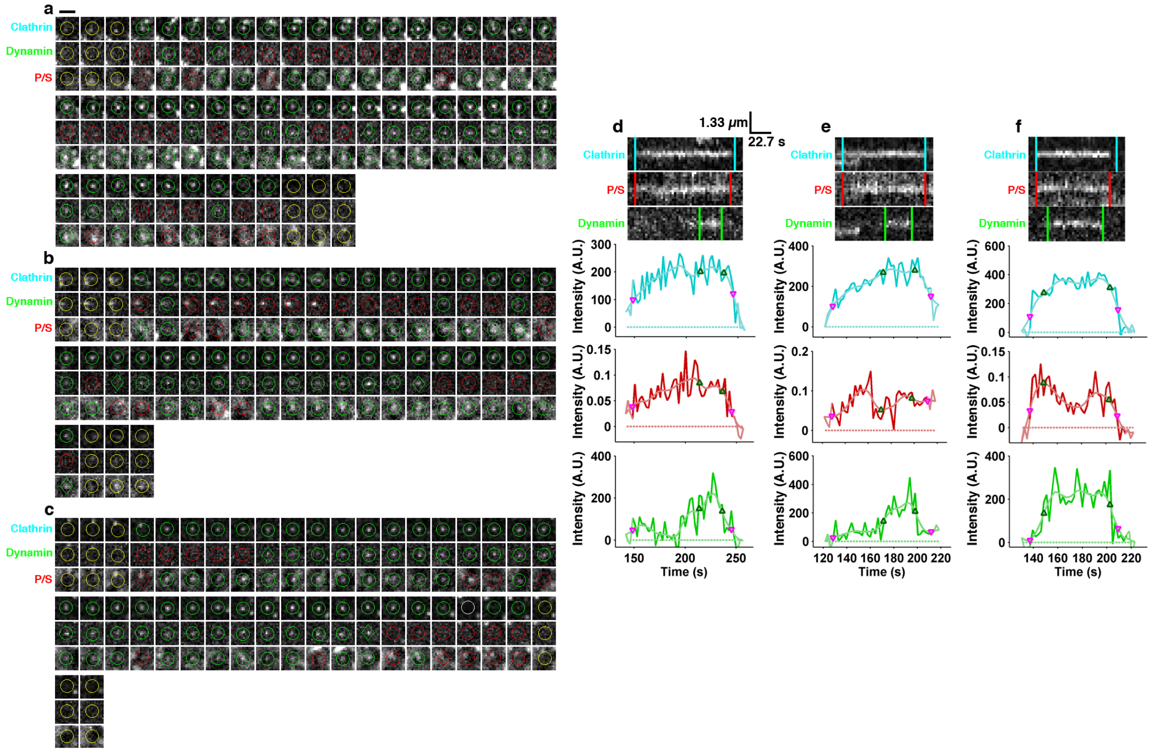

Supplementary Figure 7. Additional Class 1 CME event. a-c. Tracked and quantified results of single CME events. Clathrin-Tq2 signals are labeled with cyan, dynamin-GFP labeled in green, and P/S labeled in red. Montage centered on the clathrin tracked centroid, yellow circles indicate the 3 frame buffer before and after the tracked event. Green circles indicate a 'valid' detection, clathrin and dynamin fitted amplitude significant from the background by a t-test at $p<0.05$, and P/S SNR > 1 from the ROI-based measurement, and red circles indicate no detection. d-f. The yt-kymograph was made from the center column of pixels in the montage, and the lifetime of each component is indicated by the colored vertical bars. The local background corrected intensity for each signal is plotted with the start and stop of each lifetime indicated by the colored triangles.

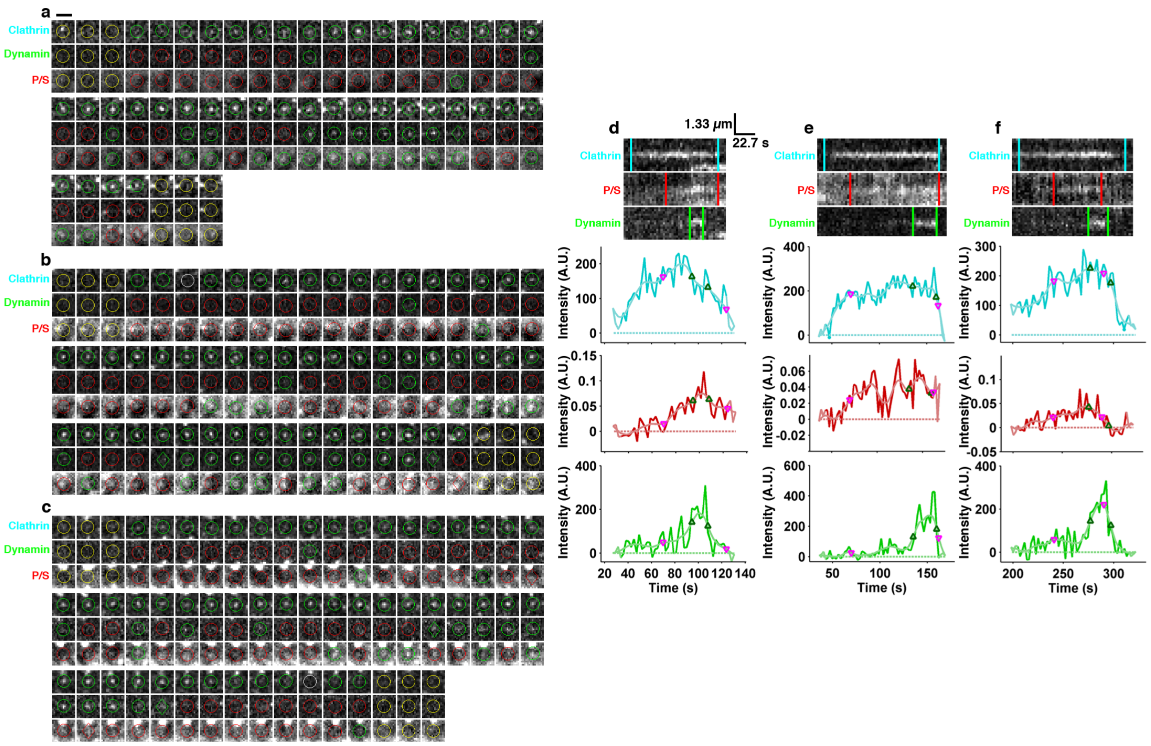

Supplementary Figure 8. Additional Class 2 CME event. a-c. Tracked and quantified results of single CME events. **d-f**. yt-kymographs and background corrected intensity traces. Lifetime of each component indicated by the colored vertical bars and colored triangles.

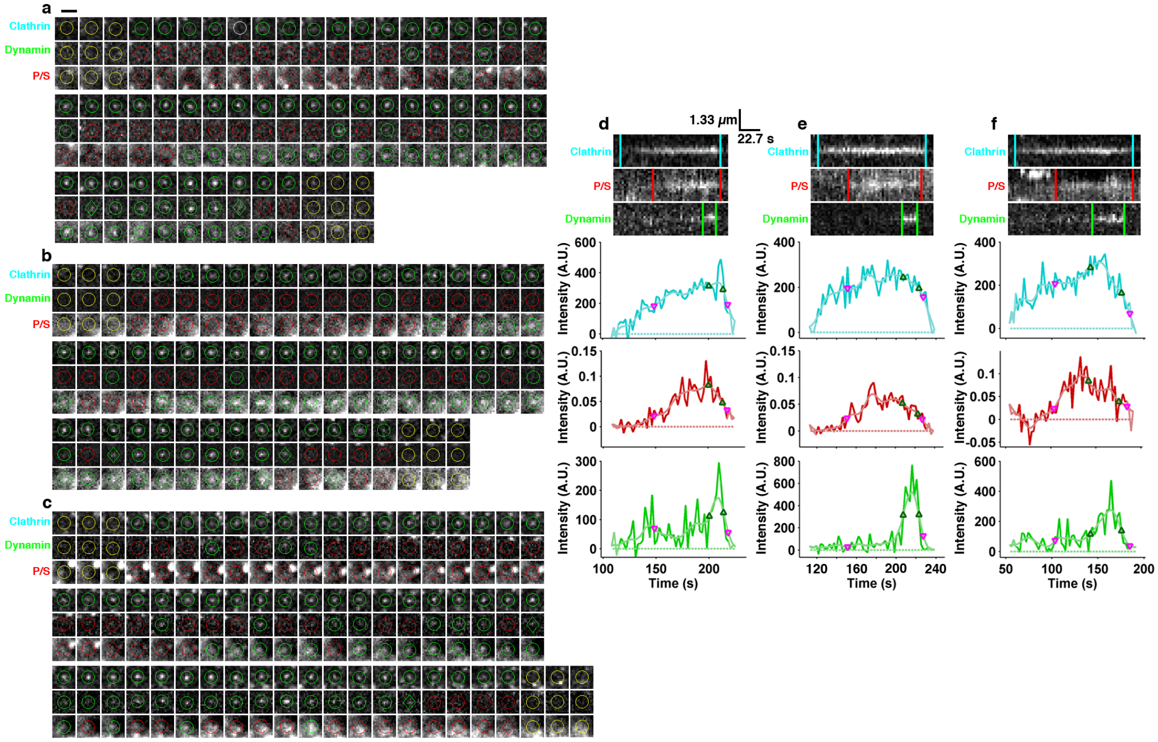

Supplementary Figure 9. Additional Class 3 CME event. a-c. Tracked and quantified results of single CME events. **d-f**. yt-kymographs and background corrected intensity traces. Lifetime of each component indicated by the colored vertical bars and colored triangles.

Supplementary Table 1. Primers for construction of pDONOR3-mTq2 vector

Bsal MCS-F	GATGGTACCAGGTGGAGACCGAATTCGGTCTCGTATGGGTGAGCAAGGGCGAGGAG
BsaI MCS-R	ATGCGGCCGCAAGCCGAGACCGGATCCGGTCTCCCTTGATTACTTGTACAGCTCGTCCAT
2A-F	GCCTGCTGAAGCAGGCTGGAGACGTGGAGGAGAACCCTGGACCTGCTAGCGGTCTCGGCTTGCGGCC
2A-R	GCCTGCTTCAGCAGGCTGAAGTTAGTAGCTCCGCTTCCCAATTGCTTGTACAGCTCGTCCATGC
Puro-NheI-F	GATCGCTAGCATGACCGAGTACAAGCCCAC
Puro-BsaI linker-R	GATGCGGCCGCAAGCCGAGACCGGATCCGGTCTCCCTTGATCAGGCACCGGGCTTGCG

Supplementary Table 2.Primers for insertion of right and left homologous are into pDONOR3-mTq2

hCLTAleft-F p202	GATGGTCTCGAGGTAGCCCGTGATGCTTGTTGGAG
hCLTAleft-R	GATGGTCTCGCATACgcttccgccgctgccaccGTGCAACAGCGGGGCCTGCTTGAG
hCLTAright-F	GATGGTCTCGCAAGAAGAGCCAACCTGTGGAAACACTACATCTGC
hCLTAright-R p205	GATGGTCTCGAAGCACTCCTGGAAACACCTGTGGTAG

Supplementary Table 3. Primers for insertion of gRNA into px330

phCas9n-CLTA-F	caccGCAGATGTAGTGTTTCCACA
phCas9n-CLTA-R	aaacTGTGGAAACACTACATCTGC

Supplementary Table 4. Primers for PCR screening

OO-CLTA-F2	attctgggctgcaccttatcaa
OO-CLTA-R2	atcacctaaaacgagccaggt
