# Supplementary material for: Membrane bending occurs at all stages of clathrin-coat assembly and defines endocytic dynamics
Source: Nat Commun. 2018 Jan 29;9:419. doi: 10.1038/s41467-018-02818-8 (PMC5789089; doi:10.1038/s41467-018-02818-8)
Supplement: Supplementary file 3 — Description of Additional Supplementary Files [file 41467_2018_2818_MOESM3_ESM.pdf]

Description of Additional Supplementary Files

File Name: Supplementary Movie 1
Description: polTIRF microscopy time lapse video of a representative cell from the pooled data in Fig. 3 with classified tracks overlaid. Left panel: clathrin-Tq2, Middle panel: dynamin-GFP, and Right panel: P/S. Track color, Green: Class 1, Red: Class 2, and Blue: Class 3. Clathrin and dynamin exposure time: 100ms, P/S exposure time: 125ms. Acquisition frame rate: 0.44 Hz (7.5 min); Frame rate, 15 fps.

File Name: Supplementary Movie 2
Description: polTIRF microscopy time lapse video of the representative kymograph in Fig. 3a, with the tracked position overlaid with green line. Left panel: clathrin-Tq2, Middle panel: dynamin-GFP, and Right panel: P/S. Imaging conditions and playback are the same as Supplementary Movie 1.
